# Porous Dielectrics in Microelectronic Wiring Applications

**DOI:** 10.3390/ma3010536

**Published:** 2010-01-18

**Authors:** Vincent McGahay

**Affiliations:** International Business Machines Corporation, 2070 Route 52, Hopewell Junction, NY 12533, USA; E-Mail: mcgahay@us.ibm.com; Tel.: +1-845-892-2055

**Keywords:** porosity, dielectric, interconnect, microelectronic, integrated circuit, semiconductor

## Abstract

Porous insulators are utilized in the wiring structure of microelectronic devices as a means of reducing, through low dielectric permittivity, power consumption and signal delay in integrated circuits. They are typically based on low density modifications of amorphous SiO_2_ known as SiCOH or carbon-doped oxides, in which free volume is created through the removal of labile organic phases. Porous dielectrics pose a number of technological challenges related to chemical and mechanical stability, particularly in regard to semiconductor processing methods. This review discusses porous dielectric film preparation techniques, key issues encountered, and mitigation strategies.

## 1. Introduction

The characteristic trend of the semiconductor industry over the last several decades has been the continual miniaturization of microelectronic devices. Integrated circuit density per unit area has doubled every one and a half to two years, a relationship commonly known as Moore’s Law. Density increases have been accompanied by comparable increases in device performance. In recent years, however, it has become necessary to introduce new technology elements in order to maintain historical trends. The appearance of porous dielectrics in microelectronic devices represents one of the significant materials changes required to keep performance improvements on pace with device density increases.

Porous dielectrics are not employed as structural components of elementary semiconductor devices such as transistors. Rather, they serve as electrical insulation between wires used to connect different devices. Their utility lies in reducing, by virtue of a lower dielectric permittivity compared to similar non-porous insulators, the capacitance between neighboring wires. This reduction has a direct influence on power consumption and, importantly for performance, the time required for an electrical signal to travel along a wire.

Excluding very low resistance interconnects, signal delay varies as the product of wire resistance *R* and capacitance *C* [1]. In early technology generations, wire *RC* delays were small compared to those associated with the operation of semiconductor devices. Thus there was little motivation for reducing either wire resistivity or the dielectric constant of the materials used to insulate wires from each other. Consequently, device interconnections in high performance parts remained for many years relatively unchanged apart from dimensional scaling and were comprised of a chemically and mechanically robust combination of aluminum-based wires embedded in amorphous SiO_2_ dielectric. The situation was more complicated for non-scaled products, in which *e.g.,* microprocessor size did not shrink due to added functionality. Such very large scale integration (VLSI) created the possibility of significant wire *RC* delays, which could be mitigated by adding wiring with large cross-sectional areas and correspondingly low resistance [2]. By the late 1990s, however, material changes were necessitated by the threat of performance improvement limitations due to wire *RC* delays that could not be adequately addressed by this approach.

The semiconductor industry’s initial response to the *RC* wiring delay problem was by-and-large to convert from aluminum-based to lower resistivity copper-based wiring [3]. This was a revolutionary development as it involved not only a completely different metallurgical system but, in many respects, fundamentally different manufacturing processes. The superior electromigration performance of copper versus aluminum wires [4] allowed copper wire heights to be reduced to match aluminum wire resistance, with the result that capacitance improvements benefitting both power and performance could be realized. Today, Cu-based wiring is industry-standard for high performance microelectronic devices. Section 2 of the review describes a production flow for fabrication of copper interconnects and considers, as a prelude to subsequent discussions, the relative impact of individual process steps on porous dielectrics in the wiring structure.

Introduction of dielectrics with reduced permittivity followed less than a decade after the conversion to copper wiring for high performance integrated circuits. A great many dielectric alternatives spanning several material classes were investigated by individual manufacturers, industry consortia such as SEMATECH, and universities. As eventually implemented in manufacturing, the progression of new dielectrics appears to have an evolutionary character: a silica-like network of bridging oxygens is maintained for structural integrity and the dielectric constant lowered through reductions in polarizability and/or density. Plasma enhanced chemical vapor deposition (PECVD) is the predominant preparation method, although other techniques such as spin-casting of polymeric solutions (called spin-on dielectrics, or SODs) exist. For the PECVD method, modification of the dielectric structure is achieved by adjusting the chemistries of the plasma precursors and process parameters such as partial pressure, flow rate, and temperature as well as electrical characteristics of the plasma. The processes developed to create lower permittivity films are not easily described as evolutionary, however, since they have involved new precursors and, for very low dielectric constants, new preparation techniques altogether.

PECVD fluorinated silica glass (FSG) films with dielectric constant in the *K* = 3.6–3.8 range compared to *K* = 4 for SiO_2_ were the first reduced permittivity insulators to appear after the introduction of copper interconnects [5]. Carbon-doped oxides (CDO, or SiCOH) with *K* = 3 and below followed next [6]. More recently, multiphase carbon-doped materials in which an organic phase is burned out either thermally, by electron-beam (e-beam) radiation, or by ultraviolet (UV) radiation to create porosity and *K* < 2.6 have been introduced [7]. Precursors used to create such labile phases are called porogens. Accordingly, the term porous is usually restricted to this class of ultra low-*K* (ULK) or pSiCOH dielectrics. This review will consider both SiCOH and pSiCOH films as the former provide the structural skeleton for the latter. Section 3 presents an overview of material preparation for different types of low-*K* and ULK dielectrics with an eye toward strategies for mechanical property optimization. Section 4 examines trends of important physical properties versus porosity level.

Although bulk physical properties are extremely important, they do not by themselves determine how well porous dielectrics perform in microelectronic applications. Surfaces which are exposed to plasma processes during manufacturing are susceptible to damage, as reflected by increased permittivity, especially as porosity increases. Section 5 discusses the issue and mitigation strategies.

Continual modification of SiCOH films to achieve high levels of porosity for capacitance reduction is recognized as posing inherent mechanical challenges for microelectronic devices. Potential solutions to this dilemma are discussed in the Concluding Remarks, Section 6.

## 2. Copper Interconnect Fabrication

The greater part of fabrication of semiconductor devices and their interconnections proceeds by wafer-level processing. A wafer starts as a slice of single crystal semiconductor, called the substrate, upon which multiple microelectronic devices are built simultaneously. This section describes schematically a process flow for wafer-level fabrication of copper interconnects, the aim of which is to identify those issues which drive mechanical optimization of low-*K* and ULK materials (discussed in Section 3) and mitigation strategies related to process-induced damage (discussed in Section 5). The fabrication method depicted is an example of what is frequently called the dual inlay process. This name refers to the fact that metal deposition occurs after both interlevel and intralevel wiring patterns have been formed in the dielectric. It is also called dual damascene, in reference to an ancient method of creating metal inlay patterns.

**Figure 1:**
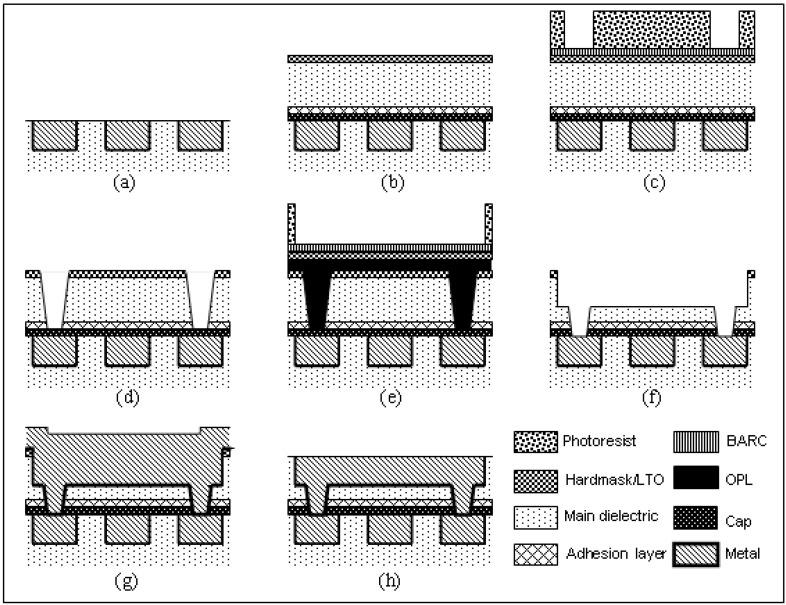
Schematic process flow for copper interconnects: (a) a planar array of wires embedded in a dielectric stack with top wire surfaces exposed; (b) deposition of a blanket dielectric stack; (c) photolithographic patterning of interlevel connections; (d) transfer of interlevel connection pattern into the dielectric stack; (e) photolithographic patterning of the intralevel wiring; (f) transfer of the intralevel wiring pattern into the dielectric stack; (g) metal deposition; (h) planarization of metal overfill.

Figure 1(a) shows a planar array of wires upon which a subsequent level of wiring is to be built. The dual inlay process begins by depositing a dielectric stack on the wafer, as illustrated in Figure 1(b). The lowermost film in the stack shown is a Cu-diffusion barrier dielectric, or cap, required for interconnect reliability [8]. An adhesion layer to improve the typically weak interfacial strength between low permittivity insulators and cap materials [9] comes next. The main (*i.e.,* low-*K* or ULK) dielectric follows and constitutes the greater part of the total stack thickness. A protective film, or hardmask, frequently SiO_2_, is employed on top of the main dielectric as a process aide, e.g., to prevent surface damage in subsequent fabrication steps.

Figure 1(c) shows the wafer after a first wiring pattern has been developed in a photo-sensitive organic material, or photoresist. The latter is typically coated over the dielectric stack following a buried anti-reflective coating (BARC), used to assist photolithographic patterning. The first wiring pattern as drawn in the figure represents interlevel connections between adjacent wiring levels. However, fabrication schemes in which the interlevel connections are patterned after intralevel connections are also possible [10].

After the interlevel wiring pattern has been printed on the wafer, the images are transferred into the dielectric stack by a reactive ion etch (RIE) process. Figure 1(d) shows the transferred pattern after any remaining photolithographic materials have been removed, or stripped, from the wafer. Stripping is a critical step insofar as processes capable of removing the organic films used in the patterning process may also damage carbon-containing low-*K* and ULK dielectrics [11].

With the interlevel connection pattern transferred into the dielectric stack, photolithographic patterning of the intralevel connections proceeds next. The main challenge at this point relates to the existence of exposed low-*K* or ULK dielectric surfaces. This raises issues of photolithographic material compatibility. Such problems can be avoided using the process structure depicted in Figure 1(e). An organic planarizing layer (OPL) is coated on the wafer followed by deposition of a low temperature oxide (LTO) film which essentially acts as a new hardmask [12]. BARC coating, photoresist coating, and printing of the intralevel wiring pattern then proceeds as for the interlevel pattern.

Figure 1(f) depicts the interconnect structure after the intralevel wiring pattern has been transferred into the dielectric stack by a RIE process, the LTO film removed, any remaining OPL material stripped from the wafer, and the cap film opened. By this point there have been many opportunities for exposed low-*K* or ULK dielectric surfaces to be damaged or contaminated by the patterning process and it is typical for the wafer to be exposed to a compatible wet clean process, e.g., dilute hydrofluoric acid [13]. As damage typically manifests itself as a densification of the exposed low-*K* film surfaces to form poor quality SiO_2_, design of the etch, strip, and clean processes is critical for maintaining low dielectric permittivity and becomes more difficult as porosity increases.

After cleaning, the wafer is ready for metallization. For copper wiring, the process begins with deposition of a barrier metal film stack, or liner, which prevents diffusion of copper into the dielectric [14]. This is important for reasons of reliability [15] and becomes more challenging as porosity in the dielectric increases, since coverage becomes difficult and the opportunity for barrier defects increases. For copper interconnects formed by electrochemical plating, as typical, the barrier film deposition process is followed by deposition of a thin Cu layer, or seed. An excess copper thickness is then plated onto the wafer, as shown in Figure 1(g). This assists the eventual planarization of the wiring level by chemical-mechanical polishing (CMP).

CMP provides one of the most critical tests of the mechanical integrity of low-*K* dielectrics. The wet environment and application of abrasive force create a situation highly conducive to crack growth, which is of particular concern for low-*K* and ULK dielectrics as these are typically in a state of tensile stress on a wafer. Moreover, the driving force for cracking can be significantly enhanced by the presence of underlying wiring [16]. Since the driving force for crack propagation increases with increasing stress level and decreasing elastic modulus, optimization of low-*K* and ULK films for these properties becomes critical.

Figure 1(h) depicts the wiring level after the CMP process has been completed. At this point the sequence starting with Figure 1(b) repeats for fabrication of the next wiring level. Complex microelectronic products can have more than ten such dual-inlay wiring levels.

## 3. Dielectric Deposition Processes

The focus of this section will be on precursor choice for property optimization of PECVD SiCOH and pSiCOH films. Background information on the PECVD technique can be found in Cote *et al.* [17] for conventional SiO_2_ films and in Grill [18] for PECVD SiCOH, pSiCOH, and other novel dielectrics such as diamond-like carbon (DLC). Fluorinated silica glass (FSG) will be briefly discussed since it involves a modification of the silica network structure—removal of bridging oxygens—that is important in carbon-doped low-*K* and ULK films.

### 3.1. Fluorinated SiO_2_

FSG films can be prepared by addition of SiF_4_ to either conventional silane (SiH_4_) or TEOS [tetraethyl orthosilicate, Si(OC_2_H_5_)_4_] based oxide deposition processes or by addition of a fluorocarbon precursor such as C_2_F_6_ to a TEOS-based deposition process [19]. At low doping levels, fluorine reduces the dielectric constant by eliminating highly polarizable silanol (SiOH) groups in the films and, at higher levels, by replacing bridging oxygen structures (Si-O-Si) in the glass network. Dielectric constant reductions down to *K* = 3 are possible. However, fluorine bonding is not stable at elevated concentrations and can adversely affect metal reliability, e.g., through attack of Ta-based barriers. FSG films as typically employed have dielectric constants in the *K* = 3.6–3.8 range. These films are not porous, although density [20] and modulus [20,21] are reduced compared to SiO_2_.

### 3.2. Low-K SiCOH

In order to achieve a dielectric constant *K*<3 in silica network films, it is necessary to modify the structure of the material in a way that reduces polarizability and/or density. This can be achieved by bonding methyl groups to Si. The elimination of bridging oxygens by this means allows the silica network to assume a less dense arrangement than in pure SiO_2_. It is possible to prepare PECVD low-*K* SiCOH films with such a structure using mixtures of hydrocarbons with the conventional oxide precursor silane [22]. More typically, silicon precursors with “built-in” methyl groups are used. These include acyclic compounds such as trimethylsilane (CH_3_)_3_SiH [23,24] and tetramethylsilane (CH_3_)_4_Si [25]. Fluorinated carbon-doped films have been prepared using mixtures of trimethylsilane and SiF_4_ [26] as well. However, cyclic precursors in which the ring structure can be preserved to some extent in the final material have been found to be among the most mechanically robust low-*K* films, a finding which has guided identification of new precursors [27,28].

 Grill [29] describes preparation of films with dielectric constant *K* = 2.8 using the cyclic compound tetramethylcyclotetrasiloxane (TMCTS, Si_4_O_4_C_4_H_16_) without use of a separate oxidizing agent. Lin, Tsui, and Vlassak [30] prepared PECVD carbon-doped films with dielectric constants in the range *K* = 3–3.3 using the cyclic compound octamethylcyclotetrasiloxane (OMCTS, C_8_H_24_O_4_Si_4_) and oxygen. They reported that the lowering of the dielectric constant was accompanied by a decrease in network Si-O bonds as detected by the FTIR peak near the 1062 cm^-1^ wavenumber and an increase in suboxide/chain Si-O bonds related to the FTIR peak near the 1023 cm^-1^ wavenumber. Such changes in bond state require careful attention as far as mechanical property optimization is concerned. Grill *et al.* [31] found that film stress decreases and that hardness and elastic modulus increase as the fraction—determined by relative FTIR peak area—of network Si-O bonds increases, with accompanying improvements in film cracking resistance. Ida *et al.* [32] found in their study of films with dielectric constants in the range *K* = 2.7–3.0 that low stress and higher mechanical strength correspond to greater cross-linking per ^13^C and ^29^Si nuclear magnetic resonance (NMR). The degree of cross-linking in low-*K* SiCOH films can be increased by exposure to UV radiation [33,34,35].

Although the plasma reactions involved in the deposition of low-*K* carbon-doped oxide films tend to be quite complex, there have been attempts to understand mechanisms and the relationship to final film properties. Tajima *et al.* [36] attempted to predict dielectric and mechanical properties of a TMCTS-based film from first principles. Although agreement was not perfect, the role of cross-linking in promoting desirable mechanical properties was confirmed. In a subsequent theoretical analysis of chemical reactions, Tajima *et al.* [37] found that precursors should promote formation of SiC_n_· radicals in the reaction chamber for efficient cross-linking.

Porosity in the low-*K* SiCOH films described in this section is interstitial in nature and not easily detectable down to *K* = 2.8 [38]. Nevertheless, these films have a relatively open structure as indicated by enhanced gas diffusivity, with reliability implications for their use in microelectronic applications [39]. Lane *et al.* [40] reported measureable porosity of 22% by volume for a film with *K* = 2.76 using positron annihilation spectroscopy (PAS/PALS). Other advanced porosimetry techniques developed to overcome difficulties associated with application of traditional gas absorption/microbalance methods to thin films include small angle X-ray scattering (SAXS) in combination with specular X-ray reflectivity (XR), small angle neutron scattering (SANS) in combination with XR, and ellipsometric porosimetry (EP) [38].

### 3.3. Ultra Low-K SiCOH

In order to achieve dielectric constants with *K* < 2.6, two-phase film deposition techniques have been developed whereby a labile organic phase is removed to leave a discrete pore structure in a more or less dense skeleton phase. Grill and Patel [41] prepared films with a dielectric constant as low as *K* = 2 by adding an organic precursor to the reaction chamber during deposition of a TMCTS-based film with nominal *K* = 2.8. The porogen phase was removed by annealing the film at 400 °C. Examples of porogen precursors that have been used with TMCTS are cyclopentene oxide (CPO, C_5_H_8_O) and butadiene monoxide (BMO, C_4_H_6_O) [42]. Gates *et al.* [43] prepared porous films using OMCTS and CPO. In principle, any hydrocarbon that can be delivered as a gas to the reaction chamber and which can be dissociated at an RF power low enough that the dielectric properties of the base film are not degraded can be used to form the porogen phase. These include cyclic unsaturated hydrocarbons, linear alkenes, and molecules with strained rings such as cycloalkene oxides [35].

The porogenic technique restores some freedom with regard to precursor choice for the skeleton phase, since this is no longer solely responsible for porosity formation, and has been extended to non-cyclic precursors accordingly. Another development has been the replacement of the thermal anneals used to remove the labile phase in early ULK films by UV radiation and e-beam assisted processes. Grill *et al.* [44] developed a *K* = 2.4 material using diethoxymethylsilane (DEMS, C_5_H_14_O_2_Si) as the skeleton precursor and bicycloheptadiene (BCHD, C_7_H_8_) as the porogen, with UV curing for removal of the labile phase. Aimaddeddine *et al.* [45] achieved *K* = 2.3 with UV-curing of an unspecified precursor system. Jousseaume *et al.* [46] compared UV and e-beam assisted removal of the labile phase in a DEMS-based film and found subtle differences in the final chemical structure.

There have been several studies aimed at understanding the formation of the dual phase and final material structure in pSiCOH films. For example, Burkey and Gleason [47] characterized condensation reactions and porogen decomposition using FTIR; Favennec *et al.* [48] studied matrix structure and porogen loading influences using FTIR and ^29^Si solid nuclear magnetic resonance; and Castex *et al.* [49] characterized precursor reaction mechanisms in porogenic films using FTIR and quadrupole mass spectroscopy.

In addition to reducing the process time required for porogen removal, radiation enhancement promotes cross-linking in the film with accompanying improvements in mechanical properties such as Young’s modulus, similar to findings in non-porous SiCOH. However, changes in stress state can be induced in other films in the dielectric stack [50] and excessive curing can actually damage pSiCOH films [51]. Multistep processes have been proposed to optimize porogen removal and mechanical properties separately [52].

The preceding observations involve UV-assisted processes but e-beam curing raises similar concerns and a particular one with regard to electrical degradation of semiconductor devices. Owada *et al.* [53] have reported a *K* = 2.25 e-beam cured film free of this issue, however.

Complexities associated with the porogen approach to ultra low-K films have sustained an interest in alternative preparation methods. Kwak *et al.* [54] describe the use of vinyltrimethylsilane [VTMS, CH_2_ = CHSi(CH_3_)_3_] for producing a porous film without introduction of a separate porogen precursor into the reaction chamber. The vinyl groups promote formation of a labile second phase which can be removed by annealing. Asami *et al.* [55] prepared a film with *K* = 2.4 using an unspecified organosilane compound containing an acetylene bond. The low dielectric constant was achieved after e-beam curing. Burkey and Gleason [56] prepared carbon-doped oxide films with dielectric constants in the *K* = 2.4–2.9 range using pulsed plasma chemical vapor deposition of both cyclic and non-cyclic organosilicon precursors with water as the oxidizing agent. They were able to achieve a hardness/dielectric constant combination for TMCTS comparable to that found for a porogen-based process. Tada *et al.* [57] prepared films with dielectric constants K<2.5 without porogens or cure processes using cyclic precursors with various side chain groups. Best results were obtained when the latter involved both vinyl (unsaturated hydrocarbon) and large alkyl (saturated hydrocarbon) groups deposited under conditions of low power and high partial pressure. Yasuhara *et al.* [58] report *K* = 2.2 films with high modulus prepared by neutral beam enhanced CVD (NBECVD) using either dimethyldiethoxysilane (DMDEOS, (CH_3_)_2_Si(OC_2_H_5_)_2_) or dimethyldimethoxysilane (DMDMOS, Si(OCH_3_)_2_(CH_3_)_2_) as precursor. In the NBECVD process, precursors are adsorbed onto the wafer and reacted using a neutral Ar beam, in contrast to conventional PECVD in which the precursors react in the plasma and subsequently deposit onto the wafer. It is evident from the preceding that several avenues to PECVD porous low-*K* materials are available. At present, however, the porogenic approach in conjunction with UV curing is the standard preparation technique for ULK films in the semiconductor industry. A detailed discussion of porous PECVD SiCOH precursor systems, preparation methods, and characterization techniques can be found in Grill [59].

### 3.4. Spin-on Dielectrics

It is possible to synthesize cage-like (RSiO_3/2_)_n_ structures, where R is hydrogen or a methyl group. The interstices at the centers of the cages can be viewed as extremely small pores. Films based on such silsesquioxane chemistries can be formed by spin-casting and drying of solutions. Furthermore, they can be converted to dense SiO_2_ by annealing and were evaluated as such as potential replacements for plasma SiO_2_ films in aluminum technologies [60]. However, it is possible to preserve the cage structure such that a film with dielectric constant at or slightly below *K* = 3 is obtained [61]. Although the preceding references pertain to hydrogen silsesquioxane (HSQ), methyl silsesquioxane (MSQ) films have also been evaluated in similar applications and form the parent material for a large class of porous spin-on dielectrics which have been evaluated for copper wiring applications.

Porous methyl silsesquioxane films can be created by removal of a labile organic phase similarly to PECVD carbon-doped films. Knoesen *et al.* [62] prepared porous MSQ films with dielectric constants in the range *K* = 1.9–2.5 using poly(dimethylaminoethyl methacrylate-co-methyl methacrylate) (DMAEMA-MMA or PMA-co-DMAEMA) as the porogen with thermal annealing. Lazzeri *et al.* [63] characterized the thermal transformation kinetics of the same system. Padovani *et al.* prepared [64] and characterized [65] porous MSQ films with dielectric constants in the range K = 2.35–2.7 in which trimethoxysilyl norbornene (TMSNB) and triethoxysilyl norbornene (TESNB) were used as porogens with thermal annealing. Chemical bonds were found between TMSNB and MSQ but not between TESNB and MSQ in the as-deposited films. Interestingly, the fracture toughness of the TESNB films was superior to the TMSNB films as well as the base MSQ material. Peng *et al.* [66] found that porogen functional groups strongly affect the final pore structure in their study of silsesquioxane-based films. As was the case for PECVD films, it is possible to create labile phases without a separate porogen precursor. Char *et al.* [67] prepared porous films by grafting various porogenic moieties onto the MSQ skeleton material.

Similar to PECVD films, radiation-assisted cures can be utilized with spin-on dielectrics. Lin *et al.* [68] found property improvements for an e-beam cured non-porogenic MSQ film used in an aluminum wiring technology. The e-beam process was found to impact semiconductor device behavior, however. Iijima *et al.* [69] compared a thermally cured porous MSQ film (*K* = 2.25) with a UV-cured MSQ film with higher carbon-content (*K* = 2.2) and improved pore structure. Volksen *et al.* [70] found that laser spike annealing helped to toughen porous organosilicate films.

Spin-on dielectrics are not limited to the silsesquioxane family of compounds, nor are they necessarily silicon-based. Wang *et al.* [71] produced a non-porogenic oxygen-free polycarbosilane-based film with *K* = 2.32, for example. Yamazaki *et al.* [72] prepared carbon-doped spin-on films with *K* = 2 using a sol-gel type method and UV curing. Silica xerogels with even lower dielectric constants have been studied as potential dielectrics for microelectronic applications [73] and used for development of porous low-*K* characterization techniques [74,75]. Integrated circuits built using spin-on organic dielectrics have also been evaluated [76,77] and porogenic versions developed [78]. High thermal expansion coefficients and related reliability issues have hindered adoption of spin-on organic films for microelectronic applications, however.

## 4. Physical Property Trends

For a given family of porous dielectrics, density is a useful indicator for other physical properties such as permittivity and elastic modulus. It is therefore a convenient starting point for examining the effect of porosity additions and some of the issues associated with its characterization. It will be apparent that direct comparison between different pSiCOH films with differing levels of porosity is difficult. It is possible to verify general trends, however.

If pores are assumed to exist as void space in an otherwise dense medium with density *ρ*_0_, porous film density decreases with increasing pore volume fraction *φ* according to the relation:

(1) $$\rho =\rho_{0}\left(1-\varphi \right)$$

This is certainly an approximation for the low-*K* dielectrics used in microelectronic applications, which lack smooth pore walls due to the interstitial character of the porosity. The density of the skeleton phase is inherently non-uniform and likely dependent on the level of porosity. These complications pertain to the assumption that *ρ*_0_ is constant in equation (1). There is also uncertainty with regard to the pore volume fraction *φ*, however. Measurement sensitivity with respect to pore size and interconnectivity can lead to differences in apparent porosity levels for a given material using different porosimetry techniques [38,79].

**Figure 2:**
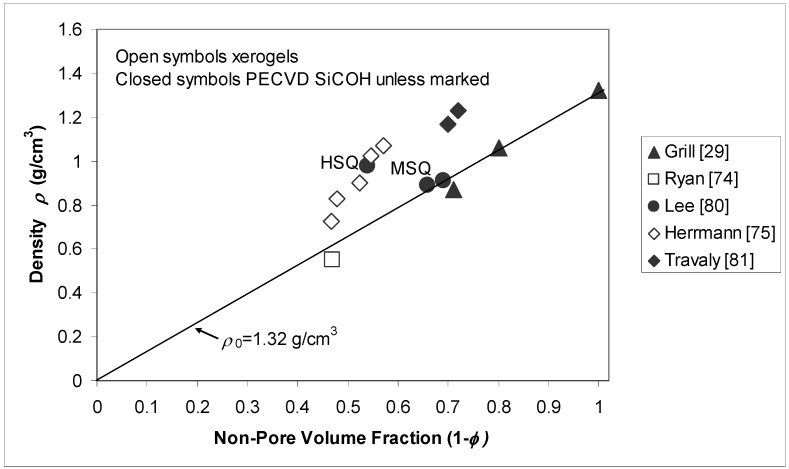
Density *ρ* plotted against non-pore volume fraction (1-*φ*) for several PECVD and spin-on dielectric materials. The line represents the prediction of equation (1) assuming *ρ*_0_ = 1.32 g/cm^3^, the density of the non-porous *K* = 2.8 film reported in [29].

Figure 2 shows a plot of film density *ρ* versus non-pore volume fraction (1- *φ*) for several PECVD SiCOH films [29,80,81], spin-on films (HSQ and MSQ) [80], and xerogels [74,75]. In general, density decreases with increasing porosity, as expected. The line indicates the prediction of equation (1) with pore wall density *ρ*_0_ = 1.32 g/cm^3^. This value equals the density of the non-porous *K* = 2.8 base film in [29] and is close to the pore wall densities for porous MSQ (*ρ*_0_ = 1.35 g/cm^3^) and porous PECVD SiCOH (*ρ*_0_ = 1.31 g/cm^3^) reported in [80]. It is evident that different values of *ρ*_0_ must apply to at least some of the films. This is confirmed by the pore wall density measurements *ρ*_0_ = 1.16 g/cm^3^ for the xerogel characterized in [74] and *ρ*_0_ = 1.83 g/cm^3^ for the porous HSQ film in [80]. It is worth noting that, although these films have been viewed as low density modifications of SiO_2_, the pore wall density cannot be assumed to be that of amorphous silica (*ρ*= 2.2 g/cm^3^). Ryan *et al.* [74] found that calculating *φ* from the measured density under this assumption gives unreasonably high predictions compared to the value determined using small angle neutron scattering.

The dielectric constant of a porous material can be calculated with the aid of the Clausius-Mossotti equation:

(2) $$\frac{K-1}{K+2}=\frac{4\pi }{3}\sum_{i}n_{i}\alpha_{i}$$

where *n_i_* is the concentration of the *i*^th^ molecular component with polarizability *α_i_*. If the molecular components exist in fixed relative proportions throughout the material then (2) can be rewritten in terms of the density *ρ*:

(3) $$\frac{K-1}{K+2}=\frac{4\pi }{3}\frac{N_{0}\alpha }{M}\rho$$

where *N*_0_ is Avogadro’s number, *M* the molecular weight, and *α* the polarizability of a stoichiometric unit of the material. Substitution of equation (1) into (3), which amounts to the effective medium approximation [82], yields:

(4) $$\frac{K-1}{K+2}=\frac{4\pi }{3}\frac{N_{0}\alpha }{M}\rho_{0}\left(1-\varphi \right)$$

The comments made in regard to *ρ*_0_ and *φ* in equation (1) apply equally well to equation (4). Validation of the relation in (4) is further complicated by the fact that stoichiometry and polarizability are highly process dependent. For example, the series of SiCOH films characterized by Lin *et al.* [30] showed decreasing values of *ρ/M* and *α* as the C/Si ratio increased and the dielectric constant decreased. Significantly, these authors found that *α* values for SiCOH films were higher than for SiO_2_, indicating that lower dipole density is the major factor in accounting for dielectric constant reduction in the former.

**Figure 3:**
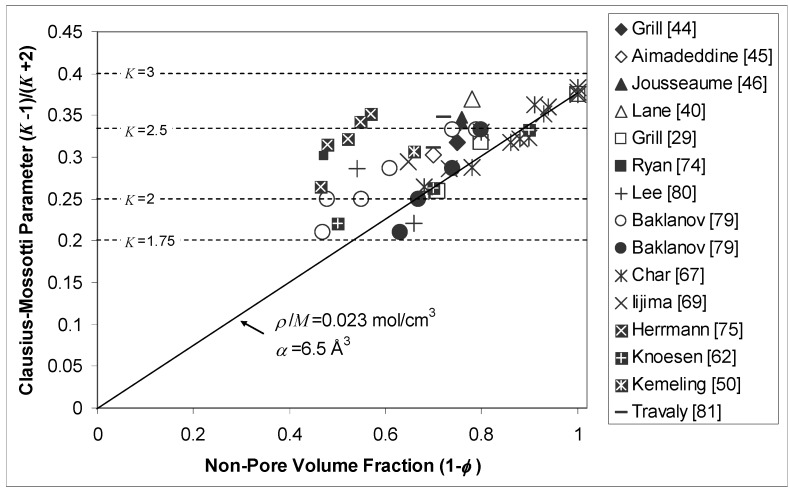
Clausius-Mossotti parameter (*K*-1)/(*K*+2) plotted against non-pore volume fraction (1-*φ*) for several PECVD and spin-on dielectric materials. The line represents the prediction of equation (4) assuming *ρ/M* = 0.023 mol/cm^3^ and *α* = 6.5 Å^3^, obtained by extrapolating data in Lin *et al.* [30] to an MSQ-like carbon-to-silicon ratio of 1:1.

Figure 3 shows a plot of the Clausius-Mossotti parameter (*K*-1)/(*K*+2) versus non-pore volume fraction (1-*φ*) for several PECVD [29,40,44,45,46,50,79,80,81] and spin-on/xerogel [62,68,69,75,79,80] films. In general, dielectric constant decreases with increasing porosity, as expected. The line indicates the prediction of equation (4) using values of *ρ/M* = 0.023 mol/cm^3^ and *α* = 6.5 Å^3^, which were extrapolated from data in Lin *et al.* [30] to an MSQ-like carbon-to-silicon ratio of 1:1. These values give a reasonable prediction of *K* for many of the films. However, it is again clear that different constants are required for different films, assuming that *φ* values are accurate. The two sets of data reported by Baklanov [79] are of particular interest in regard to this last caveat as they represent different porosity measurements on the same films. The open circles correspond to measurements made using ellipsometric porosimetry while the closed circles correspond to measurements made using small angle neutron scattering in combination with X-ray reflectance.

The dependence of Young’s modulus *E* on porosity for a one-dimensional material composed of material chains interposed with channels of open pores was derived in closed form by Wagh *et al.* [83] and predicted to follow a power law relationship with non-pore volume fraction (1- *φ*):

(5) $$E=E_{0}{\left(1-\varphi \right)}^{n}$$

The authors verified the validity of (5) in three-dimensional systems using numerical simulations. It is therefore not unreasonable to expect such a relation to hold for the porous dielectrics of interest. Munro [84] analyzed data for a large number of oxide ceramics and found the exponent *n* to range between 1.78 and 4.99, with average ~3, indicating the strength of the power law that might be expected to hold for ULK dielectrics. Difficulties similar to those encountered in analyzing the porosity dependence of density and dielectric constant apply to the case of modulus, of course, since the parameter *E*_0_, which corresponds to the modulus at zero porosity, is base material-specific and its independence with regard to porosity level questionable. In addition to the uncertainty in *φ* that exists for all parameters discussed so far, there are intrinsic measurement issues for modulus, e.g., substrate effects, which make reported values at times unreliable [59].

**Figure 4:**
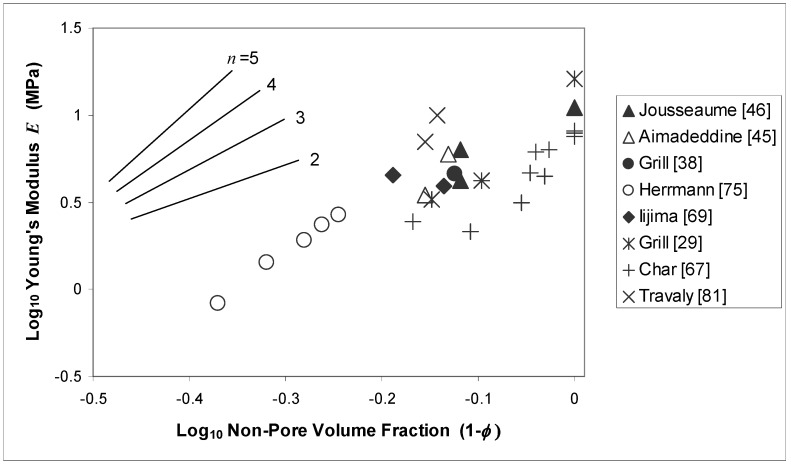
Young’s modulus *E* plotted against non-pore volume fraction (1-*φ*) for several PECVD and spin-on dielectric materials. The lines represent the expected slope for different values of exponent *n* in equation (5).

Figure 4 shows a log-log plot of Young’s modulus *E* versus non-pore volume fraction (1-*φ*) for several PECVD [29,44,45,46,81] and spin-on/xerogel [67,69,75] films. In general, modulus decreases with increasing porosity, as expected. The lines included in the figure represent the expected slope of the data for different values of the exponent *n* in equation (5). Individual fits to the larger data sets, not shown in Figure (4), indicate *n* = 4.01 for the xerogels in Herrmann [75] and *n* = 3.35 for the porous MSQ films in Char [67], which place them in the same range reported by Munro [84]. It is difficult to draw stronger conclusions, however.

The last parameter to be examined in this section is not a physical property *per se* but a process-dependent material characteristic. A crack driving force parameter, designated *G*', related to the crack extension force or fracture energy release rate [85] per unit blanket film thickness can be defined in terms of the residual film stress *σ* and elastic modulus *E* as follows:

(6) $$G'=\sigma^{2}/E$$

This parameter, which differs from the true crack driving force per unit film thickness by a multiplicative constant of order unity, provides a highly useful figure of merit for how well low-*K* and ULK films, which are generally tensile stressed, behave during CMP and other situations of mechanical loading, including service: the larger the value of *G*', the greater the risk of cohesive failure to occur. Since residual film stress is not an intrinsic physical property with a definite relationship to porosity level, it will be more convenient to consider the trend of *G*' versus dielectric constant in the discussion which follows.

**Figure 5:**
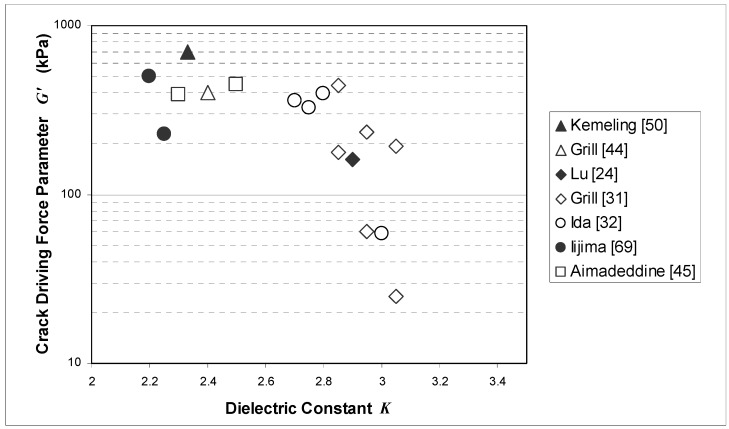
Crack driving force parameter *G*' defined in equation (6) plotted versus dielectric constant *K* for several carbon-doped oxide films including non-porous CVD SICOH, porous CVD ULK SiCOH, and porous spin-on MSQ.

Figure 5 shows a plot of crack driving force parameter *G*' versus dielectric constant *K* for several PECVD SiCOH [24,31,32], PECVD ULK SiCOH [44,45,50], and spin-on porous MSQ [69] films. The crack driving force parameter is found to increase rapidly with reduction in dielectric constant in the range *K* = 2.7–3.1 where the transition from a non-porous to porous structure occurs. With further reductions in *K*, the crack driving force appears to increase more slowly or to remain fairly flat. Irrespective of any fundamental explanation for the observed behavior, it is evident that for a given value of *K*, a wide range of crack driving forces is possible. This underscores the need for film optimization for low stress and high modulus, particularly in light of the fact that cohesive strength drops with decreasing dielectric constant [86].

It should be emphasized that the parameter *G*', while a highly useful metric, can be misleading if examined without reference to other relevant data. For example, the porous MSQ film with *K* = 2.25 studied by Iijima *et al.* [69] appears to have exceptionally good crack resistance compared to the other ULK films shown. However, as the authors indicate, this particular film has large pores which make it highly susceptible to other types of process-induced damage, discussed in the next section. The *K* = 2.2 film from the same reference has a crack driving force parameter more than twice as great but improved process robustness in comparison, due to its finer pore structure.

## 5. Plasma-Induced Damage and Repair

The discussion of the preceding section focused on optimization of low-*K* and ULK dielectric deposition processes for bulk physical properties. However, as mentioned in Section 2, the exposure of the dielectric to reactive ion etch and strip processes required for interconnect patterning can damage the film such that the original bulk properties, dielectric constant in particular, are severely degraded. The propensity for damage is exacerbated by the presence of porosity which allows deeper penetration of the plasma into the film, especially for highly interconnected pores [87]. Worsley *et al.* [88] showed that solvent penetration studies are effective for characterizing the interconnectedness of pore morphology. Solvent diffusivity was found to increase rapidly at about 25% pore volume, indicating the onset of open porosity. The data in Figure 3 suggest that this increased sensitivity to plasma penetration and damage occurs as the dielectric constant drops below *K* = 2.5 approximately.

Strategies involving modified porogen removal approaches have been developed for reducing plasma-induced damage in ULK films. Kagawa *et al.* [89] used partial porogen removal after ULK film deposition with removal of remaining porogen by thermal treatment after wires had been fully formed, a variant on full post-metallization porogen removal [90]. These approaches require that the porogen phase be stable at normal process temperatures prior to final removal [91]. Retention of the porogen phase evidently blocks penetration of the plasma into the film. A similar benefit is seen in the conventional porogen removal approach by increasing carbon content in the post-cured film [92].

Considerable care is required in developing etch and strip chemistries. During pattern transfer, dimensional control is typically achieved by depositing a thin fluorocarbon film on the sidewalls of the dielectric to prevent lateral etching. This film helps to limit damage to the dielectric but must be removed to avoid metallization issues. The challenge is to find strip chemistries which are effective at removing the fluorocarbon film and any remaining sacrificial patterning films such as OPL with minimal damage to the dielectric. Furukawa *et al.* in studies of porous spin-on MSQ [93] and CVD SiCOH [94] films found that CF_4_/O_2_ and N_2_/O_2_ strip chemistries were highly effective at removing fluorocarbon but caused extensive depletion of carbon in the etched films, while N_2_/H_2_ chemistries had thinner damage layers but were less effective at fluorine removal. Aimadeddine *et al.* [95] reported that oxygen-based strips had deleterious effects and nitrogen-based strips positive effects on interconnect reliability. Dalton *et al.* [96] found that oxidizing chemistries were highly damaging in porous films as well, although viable in dense films. Positive results for an oxidizing strip chemistry with ULK were reported, however, by Shi *et al.* [97] and Liu *et al.* [98] for CO_2_-based strip processes. N_2_/H_2_ strips have been recommended for SiO_2_ aerogels [99] and for post-metallization porogen removal schemes [100].

Apparently contradictory data abound in the literature and reflect the fact that development of etch and strip process is very much an art, with strip performance being highly material dependent. In contrast to the several positive results cited above for reducing chemistries, Grill and coworkers found that H_2_ containing plasmas were highly damaging to PECVD ULK SiCOH [101], with the extent of the damage influenced by the precursor system of the dielectric [102]. Gas mixture composition in the strip process matters as well. Grill and Patel [103] found that N_2_/H_2_ was superior to N_2_/He but inferior to NH_3_ with respect to damage propensity.

Worsley *et al.* [104] found that different strip processes tend to result in similar final damage signatures (e.g., loss of methyl groups, increased cross linking and densification with concomitant increases in dielectric constant) but differ in kinetics. Ion bombardment appears to be an important factor [11]. Downstream plasma strip processes, in which the wafer is remote from the plasma source, appear to reduce damage propensity [11,105].

No strip process appears to be completely satisfactory at achieving a damage-free structure in ULK materials and corrective actions are typically required. A conventional approach is to try to limit the damage as much as possible and to remove the damaged layer prior to metallization, e.g., by dilute hydrofluoric acid (DHF) [13]. Broussous *et al.* [106] found that a DHF clean was necessary to improve interconnect yield but had a narrow process window.

An alternative approach is to intentionally damage the exposed surfaces in a controlled manner in order to create a permanent thin dense layer [107,108,109]. NH_3_ plasma pore sealing has been found to prevent moisture uptake in ULK materials [110] and even to be resistant to limited DHF exposure [111]. The disadvantage of plasma pore sealing is that it tends to raise the effective dielectric constant, although low impact processes have been reported [112]. A potential advantage is mitigation of metal barrier coverage issues over porous surfaces [113]. Pore sealing techniques based on organic polymer deposition [114,115] have also been investigated. These do not address plasma-induced damage, however.

A third alternative is to try to restore the ULK dielectric constant by damage repair processes, frequently called silylation. Supercritical CO_2_ can be used to carry various organosilicon compounds into the interior of a damaged ULK film for removal of silanol (SiOH) groups [116,117]. Hexamethyldisilazane (HMDS) [118] and TMCTS [119] treatments has been reported to have similar damage repair capability. Without the assistance of supercritical CO_2_, the ability for repair chemicals to enter the damaged dielectric depends heavily on the surface tension state [120].

Yet another, perhaps counterintuitive, approach is to use a plasma for film recovery. Bao *et al.* [121] reported the ability of a CH_4_ plasma to repair oxygen plasma damage, although the recovery was not complete due to the formation of a thin polymer film on exposed surfaces, which limited penetration.

## 6. Concluding Remarks

The aim of this review was five-fold: to describe the microelectronic scaling trends which led to the introduction of porous insulators in integrated circuits; to highlight the manufacturing process sensitivities which dictate the physical property requirements for these materials; to identify the characteristics of material precursors which help to promote the required properties; to validate the expected dependencies of important physical properties on porosity level; and to describe methods for guaranteeing the desired physical properties in the final integrated wiring structure. It cannot claim to be exhaustive in terms of all relevant contributions to the literature, particularly in regard to the vast amount of characterization work documented. Nor is it completely comprehensive with respect to all issues relevant to low-*K* and porous dielectrics in microelectronic applications, e.g., protection of the wiring structure during chip dicing [122,123] and in service [124,125].

The review concludes with some final remarks on possible future directions for very low permittivity dielectrics in microelectronic applications. There are essentially three paths available. The first is to continue to increase porosity levels using established ULK dielectric preparation techniques. This will require careful optimization of the skeletal structure for mechanical properties. Recent work testifies to the viability of this approach. Adjustment of precursor chemistries to promote formation of alkyl bridge groups, for example, shows promise for both PECVD [126] and spin-on films [127]. The second is to develop new materials with the desired structure/property relations. Periodic porous self-assembled SiO_2_ films, for example, show improved mechanical properties versus random structures [128], although pore size control is still critical [129]. The third is to adopt a paradigm shift for achieving low effective dielectric constants. Airgaps, in which low permittivity areas are created in critical areas with the majority of the structure remaining a robust, higher-*K* film [130] are an especially attractive solution to problem of achieving low-*K* with superior mechanical properties, as indicated by an already extensive literature.

## References

[1] Taur Y., Chandrakasan A., Bowhill W.J., Fox F. (2001). CMOS Scaling and Issues in Sub-0.25 um Systems. Design of High Performance Microprocessor Circuits.

[2] Taur Y., Ning T. (2009). Fundamentals of Modern VLSI Devices.

[3] Edelstein D., Heidenreich J., Goldblatt R., Cote W., Uzoh C., Lustig N., Roper P., McDevitt T., Motsiff W., Simon A., Dukovic J., Wachnik R., Rathore H., Schulz R., Su L., Luce S., Slattery J. (1997). Full Copper Wiring in a Sub-0.25 µm CMOS ULSI Technology. International Electron Devices Meeting 1997. IEDM Technical Digest (Cat. No.97CH36103).

[4] Hu C.K., Luther B. (1995). Electromigration in 2-level interconnects of Cu-alloy and Al-alloy. Mater. Chem. Phys..

[5] Leobandung E., Barth E., Sherony M., Lo S.-H., Schulz R., Chu W., Khare M., Sadana D., Schepis D., Bolam R., Sleight I., White F., Assaderaghi F., Moy D., Biery G., Goldblan R., Chen T.-C., Davari B., Shahidi G. (1999). High performance 0.18 μm SOI CMOS technology. International Electron Devices Meeting 1999. Technical Digest (Cat. No.99CH36318).

[6] Edelstein D., Davis C., Clevenger L., Yoon M., Cowley A., Nogami T., Rathore H., Agarwala B., Arai S., Carbone A., Chanda K., Cohen S., Cote W., Cullinan M., Dalton T., Das S., Davis P., Demarest J., Dunn D., Dziobkowski C., Filippi R., Fitzsimmons J., Flaitz P., Gates S., Gill J., Grill A., Hawken D., Ida K., Klaus D., Klymko N., Lane M., Lane S., Lee J., Landers W., Li W.-K., Lin Y.-H., Liniger E., Liu X.-H., Madan A., Malhotra S., Martin J., Molis S., Muzzy C., Nguyen D., Nguyen S., Ono M., Parks C., Questad D., Restaino D., Sakamoto A., Shaw T., Shimooka Y., Simon A., Simonyi E., Tempest S., Van Kleeck T., Vogt S., Wang Y.-Y., Wille W., Wright J., Yang C.-C., Ivers T. (2004). Reliability, Yield, and Performance of a 90 nm SOI/Cu/SiCOH Technology. Proceedings of the IEEE 2004 International Interconnect Technology Conference (IEEE Cat. No.04TH8729).

[7] Sankaran S., Arai S., Augur R., Beck M., Biery G., Bolom T., Bonilla G., Bravo O., Chanda K., Chae M., Chen F., Clevenger L., Cohen S., Cowley A., Davis P., Demarest J., Doyle J., Dimitrakopoulos C., Economikos L., Edelstein D., Farooq M., Filippi R., Fitzsimmons J., Fuller N., Gates S.M., Greco S.E., Grill A., Grunow S., Hannon R., Ida K., Jung D., Kaltalioglu E., Kelling M., Ko T., Kumar K., Labelle C., Landis H., Lane M.W., Landers W., Lee M., Li W., Liniger E., Liu X., Lloyd J.R., Liu W., Lustig N., Malone K., Marokkey S., Matusiewicz G., McLaughlin P.S., McLaughlin P.V., Mehta S., Melville I., Miyata K., Moon B., Nitta S., Nguyen D., Nicholson L., Nielsen D., Ong P., Patel K., Patel V., Park W., Pellerin J., Ponoth S., Petrarca K., Rath D., Restaino D., Rhee S., Ryan E.T., Shoba H., Simon A., Simonyi E., Shaw T.M., Spooner T., Standaert T., Sucharitaves J., Tian C., Wendt H., Werking J., Widodo J., Wiggins L., Wisnieff R., Ivers T. (2006). 45 nm CMOS node Cu/low-k/ ultra low-k PECVD SiCOH (k = 2.4) BEOL technology. 2006 International Electron Devices Meeting (IEEE Cat No. 06CH37807C).

[8] Wang T.C., Cheng Y.L., Wang Y.L., Hsieh T.E., Hwang G.J., Chen C.F. (2006). Comparison of characteristics and integration of copper diffusion-barrier dielectrics. Thin Solid Films.

[9] Grill A., Edelstein D., Lane M., Patel V., Gates S., Restaino D., Molis S. (2008). Interface engineering for high interfacial strength between SiCOH and porous SiCOH interconnect dielectrics and diffusion caps. J. Appl. Phys..

[10] Kriz J., Angelkort C., Czekalla M., Huth S., Meinhold D., Pohl A., Schulte S., Thamm A., Wallace S. (2008). Overview of dual damascene integration schemes in Cu BEOL integration. Microelectron. Eng..

[11] Fuller N.C.M., Worsley M.A., Nitta S., Dalton T., Tai T.L., Bent S., Magbitang T., Dubois G., Miller R., Volksen W., Sankar M., Purushothoman S. (2006). Analysis of plasma-induced modification of ULK and eULK materials: dual damascene processing challenges for 45nm (κ < 2.4) and beyond BEOL technologies. Proceedings of the IEEE 2006 International Interconnect Technology Conference (IEEE Cat. No. 06TH8862C).

[12] Cote W., Edelstein D., Bunke C., Biolsi P., Wille W., Baks H., Conti R., Dalton T., Houghton T., Li W.-K., Lin Y.-H., Moskowitz S., Restaino D., Van Kleeck T., Vogt S., Ivers T., Russell S.W., Mills M.E., Osaki A., Yoda T. (2007). Non-poisoning dual Damascene patterning scheme for low-k and ultra low-k BEOL. Advanced Metallization Conference 2006 (AMC 2006). Proceedings.

[13] Le Q.T., Baklanov M.R., Kesters E., Azioune A., Struyf H., Boullart W., Pireaux J.J., Vanhaelemeersch S. (2005). Removal of plasma-modified low-k layer using dilute HF: Influence of concentration. Electrochem. Solid State Lett..

[14] Holloway K., Fryer P.M., Cabral C., Harper J.M.E., Bailey P.J., Kelleher K.H. (1992). Tantalum as a diffusion barrier between copper and silicon – failure mechanism and effect of nitrogen additions. J. Appl. Phys..

[15] Lloyd J.R., Ponoth S., Liniger E., Cohen S. (2007). Role of Cu in TDDB of low-k dielectrics. 2007 IEEE International Reliability Physics Symposium Proceedings (IEEE Cat. No.07CH37867).

[16] Liu X.H., Shaw T.M., Lane M.W., Rosenberg R.R., Lane S.L., Doyle J.P., Restaino D., Vogt S.F., Edelstein D.C. (2004). Channel cracking in low-k film son patterned multilayers. Proceedings of the IEEE 2004 International Interconnect Technology Conference.

[17] Cote D.R., Nguyen S.V., Stamper A.K., Armbrust D.S., Tobben D., Conti R.A., Lee G.Y. (1999). Plasma-assisted chemical vapor deposition of dielectric thin films for ULSI semiconductor circuits. IBM J. Res. Develop..

[18] Nguyen S.V. (1999). High-density plasma chemical vapor deposition of silicon-based dielectric films for integrated circuits. IBM J. Res. Develop..

[19] Shapiro M.J., Nguyen S.V., Matsuda T., Dobuzinsky D. (1995). CVD of fluorosilicate glass for ULSI applications. Thin Solid Films.

[20] Sikder A.K., Kumar A., Thagella S., Yota J. (2004). Effects of properties and growth parameters of doped and undoped silicon oxide films on wear behavior during chemical mechanical planarization process. J. Mater. Res.

[21] Oshima T., Tamaru T., Ohmori K., Aoki H., Ahihara H., Saito T., Yamaguchi H., Miyauchi M., Torii K., Murata J., Satoh A., Miyazaki H., Hinode K. (2000). Improvement of thermal stability of via resistance in dual damascene copper interconnection. International Electron Devices Meeting 2000. Technical Digest. IEDM (Cat. No.00CH37138).

[22] Grill A., Patel V. (1999). Low dielectric constant films prepared by plasma-enhanced chemical vapor deposition from tetramethylsilane. J. Appl. Phys..

[23] Loboda M.J. (2000). New solutions for intermetal dielectrics using trimethylsilane-based PECVD processes. Microelectron. Eng..

[24] Lu J.C., Chang W., Jang S.M., Yu C.H., Liang M.S. (2004). Development of 300mm low K dielectric for 0.13 um BEOL damascene process. Proceedings of the IEEE 2004 International Interconnect Technology Conference (IEEE Cat. No.04TH8729).

[25] Shamiryan D., Weidner K., Gray W.D., Baklanov M.R., Vanhaelemeersch S., Maex K. (2002). Comparative study of PECVD SiCOH low-k films obtained at different deposition conditions. Microelectron. Eng..

[26] Cheng Y.L., Wang Y.L., Hwang G.J., Lee W.H., O'Neill M.L., Tang A., Wu C.L. (2007). Optimization and integration of trimethylsilane-based organosilicate glass and organofluorinated silicate glass dielectric thin films for Cu damascene process. J. Vac. Sci. Technol. B.

[27] Grill A., Perraud L., Patel V., Jahnes C., Cohen S. (1999). Low dielectric constant SiCOH films as potential candidates for interconnect dielectrics. Low-Dielectric Constant Materials V. Proceedings (Materials Research Society Symposium Proceedings).

[28] Rouessac V., Favennec L., Remiat B., Jousseaume V., Passernard G., Durand J. (2005). Precursor chemistry for ULK CVD. Microelectron. Eng..

[29] Grill A. (2003). Plasma enhanced chemical vapor deposited SiCOH dielectrics: from low-k to extreme low-k interconnect materials. J. Appl. Phys..

[30] Lin Y., Tsui T.Y., Vlassak J.J. (2006). Octomethylcyclotetrasiloxane-based, low-permittivity organosilicate coatings. J. Electrochem. Soc..

[31] Grill A., Edelstein D., Restaino D., Lane M., Gates S., Liniger E., Shaw T., Liu X.H., Klaus D., Patel V., Cohen S., Simonyi E., Klymko N., Lane S., Ida K., Vogt S., Van Kleeck T., Davis C., Ono M., Nogami T., Ivers T. (2004). Optimization of SiCOH dielectrics for integration in a 90 nm CMOS technology. Proceedings of the IEEE 2004 International Interconnect Technology Conference (IEEE Cat. No.04TH8729).

[32] Ida K., Nguyen S., Lane S., Klymko N., Chanda K., Chen F., Christiansen C., Cohen S., Cullinan M., Dziobkowski C., Flaitz P., Fitzsimmons J., Fukasawa M., Gill J., Grill A., Inoue K., Kumar K., Labelle C., Lane M., Liniger E., Madon A., Malone K., Martin J., McGahay V., Minami M., Molis S., Restaino D., Sakamoto A., Sankar M., Sherwood M., Simonyi E., Shimooka Y., Tai L., Widodo J., Wildman H., Ono M., McHerron D., Nye H., Edelstein D., Nogami T., Ivers T., Brongersma S.H., Taylor T.C., Tsujimura M., Masu K. (2006). PECVD low-k (~2.7) dielectric SiCOH film development and integration for 65nm CMOS devices. Advanced Metallization Conference 2005 (AMC 2005). Proceedings of the Conference.

[33] Prager L., Marsik P., Wennrich L., Baklanov M.R., Naumov S., Pistol L., Schneider D., Gerlach J.W., Verdonck P., Buchmeiser M.R. (2008). Effect of pressure on efficiency of UV curing of CVD-derived low-k material at different wavelengths. Microelectron. Eng..

[34] Gage D.M., Guyer E.P., Stebbins J.F., Cui Z., Al-Bayati A., Demos A., MacWilliams K.P., Dauskardt R.H. (2006). UV curing effects on glass structure and mechanical properties of organosilicate low-k thin films. Proceedings of the IEEE 2006 International Interconnect Technology Conference (IEEE Cat. No. 06TH8862C).

[35] Chapelon L.L., Vitiello J., Gonchond J.P., Barbier D., Torres J. (2006). UV curing effects on mechanical and electrical performance of a PECVD non-porogen SiOC:H films (in k [2.2–2.4] range) for 45nm node and below. Microelectron. Eng..

[36] Tajima N., Ohno T., Hamada T., Yoneda K., Kobayashi N., Shinriki M., Miyazawa K., Sakota K., Hasaka S., Inoue M. (2006). Carbon-rich SiCOH films with hydrocarbon network bonds for low-k dielectrics: first-principles investigation. Proceedings of the IEEE 2006 International Interconnect Technology Conference (IEEE Cat. No. 06TH8862C).

[37] Tajima N., Ohashi Y., Nagano S., Xu Y., Matsumoto S., Kada T., Ohno T. (2009). Theoretical analyses of chemical reactions for forming hydrocarbon-bridged SiCOH low-K films in PECVD processes. 2009 IEEE International Interconnect Technology Conference–IITC.

[38] Grill A., Patel V., Rodbell K.P., Huang E., Baklanov M.R., Mogilnikov K.P., Toney M., Kim H.C. (2003). Porosity in plasma enhanced chemical vapor deposited SiCOH dielectrics: a comparative study. J. Appl. Phys..

[39] Shaw T.M., Jimerson D., Haders D., Murray C.E., Grill A., Edelstein D.C., Chidambarrao D., Ray G.W., Smy T., Ohla T., Tsujimura M. (2004). Moisture and oxygen uptake in low k/copper interconnect structures. Advanced Metallization Conference 2003 (AMC 2003).

[40] Lane S., Fukusawa M., Angyal M., Chanda K., Chen F., Christiansen C., Fitzsimmons J., Gill J., Ida K., Inoue K., Kumar K., Li B., McLaughlin P., Melville I., Nimami M., Nguyen S., Penny C., Sakamoto A., Shimooka Y., Ono M., McHerron D., Nogami T., Ivers T., Brongersma S.H., Taylor T.C., Tsujimura M., Masu K. (2006). BEOL process integration with Cu/SiCOH (k = 2.8) low-k interconnects at 65nm groundrules. Advanced Metallization Conference 2005 (AMC 2005). Proceedings of the Conference.

[41] Grill A., Patel V. (2001). Ultralow-k dielectrics prepared by plasma-enhanced chemical vapor deposition. Appl. Phys. Lett..

[42] Grill A., Patel V. (2008). Ultralow dielectric constant pSiCOH films prepared tetramethylcyclo-tetrasiloxane as skeleton precursor. J. Appl. Phys..

[43] Gates S.M., Neumayer D. A., Sherwood M. H., Grill A., Wang X., Sankarapandian M. (2007). Preparation and structure of porous dielectrics by plasma enhanced chemical vapor deposition. J. Appl. Phys..

[44] Grill A., Gates S., Dimitrakopoulos C., Pagel V., Cohen S., Ostrovski Y., Liniger E., Simonyi E., Restaino D., Sankaran S., Reiter S., Demos A., Yim K.S., Nguyen V., Rocha J., Ho D. (2008). Development and optimization of porous pSiCOH interconnect dielectrics for 45 nm and beyond. Proceedings of the IEEE 2008 International Interconnect Technology Conference.

[45] Aimadeddine M., Jousseaume V., Amal V., Favennec L., Farcy A., Zenasni A., Assous M., Vilmay M., Jullian S., Maury P., Delaye V., Jourdan N., Vanypre T., Brun P., Imbert G., LeFriec Y., Mellier M., Chaabouni H., Chapelon L.L., Hamioud K., Volpi F., Louis D., Passemard G., Torres J. (2007). Robust integration of an ULK SiOCH dielectric (k = 2.3) for high performance 32 nm node BEOL. 2007 10th International Interconnect Technology Conference.

[46] Jousseaume V., Zenasni A., Favennec L., Gerbaud G., Bardet M., Simon J.P., Humbert A. (2007). Comparison between e-beam and ultraviolet curing to perform porous a-SiOC:H. J. Electrochem. Soc..

[47] Burkey D.D., Gleason K.K. (2004). Temperature-resolved Fourier transform infrared study of condensation reactions and porogen decomposition in hybrid organosilicon porogen films. J. Vac. Sci. Technol. A.

[48] Favennec L., Jousseaume V., Gerbaud G., Zenasni A., Passemard G. (2007). Ultralow k using a plasma enhanced chemical vapor deposition porogen approach: matrix structure and porogen loading influences. J. Appl. Phys..

[49] Castex A., Jousseaume V., Deval J., Bruat J., Favennec L., Passemard G. (2008). Ultralow k using a plasma-enhanced chemical vapor deposition porogen approach: study of the precursor reaction mechanisms. J. Vac. Sci. Technol. A.

[50] Kemeling N., Matsushita K., Tsuji N., Kagami K., Kato M., Kaneko S., Sprey H., de Roest D., Kobayashi N. (2007). A robust k~2.3 SiCOH low-k film formed by porogen removal with UV-cure. Microelectron. Eng..

[51] Nakao S.-I., Ushio J., Ohno T., Hamada T., Kamigaki Y., Kato M., Yoneda K., Kondo S., Kobayashi N. (2006). UV/EB cure mechanism for porous PECVD/SOD low-k SiCOH materials. Proceedings of the IEEE 2006 International Interconnect Technology Conference (IEEE Cat. No. 06TH8862C).

[52] Seo K., Oka Y., Nomura K., Tsutsue M., Kobori E., Goto K., Mizukami Y., Ohtsuka T., Tsukamoto K., Matsumoto S., Ueda T. (2009). New multi-step UV curing process for porogen-based porous SiOC. 2009 IEEE International Interconnect Technology Conference – IITC.

[53] Owada T., Ohara N., Watatani H., Kouno T., Kudo H., Ochimizu H., Sakoda T., Asami N., Ohkura Y., Fukuyama S., Tsukune A., Nakaishi M., Nakamura T., Nara Y., Kase M. (2009). Advanced BEOL integration using porous low-k (k = 2.25) material with charge-damage-less electron beam cure technique. 2009 IEEE International Interconnect Technology Conference – IITC.

[54] Kwak S.-K., Jeong K.H., Rhee S.W. (2004). Nanocomposite low-k SiCOH films by direct PECVD using vinyltrimethylsilane. J. Electrochem. Soc..

[55] Asami N., Owada T., Akiyama S., Ohara N., Iba Y., Kouno T., Kudo H., Takesako S., Osada T., Kirimura T., Watatani H., Uedono A., Nara Y., Kase M. (2009). Novel low-k SiOC (k = 2.4) with superior tolerance to direct polish and ashing for advanced BEOL integration. 2009 IEEE International Interconnect Technology Conference – IITC.

[56] Burkey D.D., Gleason K.K. (2004). Organosilicon thin films deposited from cyclic and acyclic precursors using water as an oxidant. J. Electrochem. Soc..

[57] Tada M., Yamamoto H., Ito F., Takeuchi T., Furutake N., Hayashi Y. (2005). Chemical structure effects of ring-type siloxane precursors on properties of plasma-polymerized porous SiCOH films. J. Electrochem. Soc..

[58] Yasuhara S., Chung J., Tajima K., Yano H., Kadomura S., Yoshimaru M., Matsunaga N., Kubota T., Ohtake H., Samukawa S. (2009). Structure-designable method to form super low-k SiOC film (k = 2.2) by neutral-beam-enhanced chemical vapour deposition. J. Phys. D–Appl. Phys..

[59] Grill A. (2009). Porous pSiCOH ultralow-k dielectrics for chip interconnects prepared by PECVD. Annu. Rev. Mater. Res..

[60] Pineda R., Chiang C., Fraser D.B. (1990). A new caged structure spin-on silica for multilevel interconnect application. 1990 Proceedings. Seventh International IEEE VLSI Multilevel Interconnection Conference (Cat. No.90TH0325–1).

[61] McGahay V., Acovic A., Agarwala B., Endicott G., Nguyen D., Shapiro M., Yankee S. Process integration and reliability of hydrogen silsesquioxane in direct-on-metal application. 1996 Proceedings Thirteenth International VLSI Multilevel Interconnection Conference (VMIC).

[62] Knoesen A., Song G., Volksen W., Huang E., Magbitang T., Sundberg L., Hedrick J.L., Hawker C.J., Miller R.D. (2004). Porous organosilicates low-dielectric films for high frequency devices. J. Electron. Mater..

[63] Lazzeri P., Vanzetti L., Anderle M., Bersani M., Park J.J., Lin Z., Briber R.M., Rubloff G.W., Kim H.C., Miller R.D. (2005). Thin-film transformations and volatile products in the formation of nanoporous low-k polymethylsilsesqioxane-based dielectric. J. Vac. Sci. Technol. B.

[64] Padovani A.M., Rhodes L., Allen S.A.B., Kohl P.A. (2002). Chemically bonded porogens in methylsilsesquioxane I. Structure and bonding. J. Electrochem. Soc..

[65] Padovani A.M., Riester L.K., Rhodes L., Allen S.A.B., Kohl P.A. (2002). Chemically bonded porogens in methylsilsesquioxane II. Electrical, optical, and mechanical properties. J. Electrochem. Soc..

[66] Peng H.-G., Vallery R.S., Liu M., Frieze W.E., Gidley D.W., Yim J.-H., Kim J. (2005). Deducing nonopore structure and growth mechanisms in porogen-templated silsesquioxane thin films. Appl. Phys. Lett..

[67] Char K., Cha B.J., Kim S. (2004). Material issues for nanoporous ultra low-k dielectrics. Proceedings of the IEEE 2004 International Interconnect Technology Conference (IEEE Cat. No.04TH8729).

[68] Lin C.-F., Tung I-C., Feng M.-S. (1999). Effects of methyl silsesquioxane electron-beam curing on device characteristics of logic and four-transistor static random-access memory. Jpn. J. Appl. Phys. Pt. 1.

[69] Iijima T., Lin Q., Chen S., Labelle C., Fuller N., Ponoth S., Cohen S., Lloyd J., Dunn D., Muzzy C., Gill J., Nitta S., McGahay V., Tyberg C., Spooner T., Nye H. (2006). BEOL integration of highly damage-resistant porous ultra low-K material using direct CMP and via-first process. Proceedings of the IEEE 2006 International Interconnect Technology Conference (IEEE Cat. No. 06TH8862C).

[70] Volksen W., Dubois G., Kellock A., Magbitang T.P., Miller R.D., Cohen S., Simonyi E., Ramirez L., Wang Y. (2006). Laser spike annealing: a novel post-porosity treatment for significant toughening of low-k organosilicates. Proceedings of the IEEE 2006 International Interconnect Technology Conference (IEEE Cat. No. 06TH8862C).

[71] Wang P.-I., Wu Z., Lu T.-M., Interrante L. (2006). A novel polycarbosilane-based low-k dielectric material. J. Electrochem. Soc..

[72] Yamazaki T., Hirakawa M., Nakayama T., Murakami H. (2009). Development of porous silica ultra low-k films for 32nm-node interconnects and beyond. 2009 IEEE International Interconnect Technology Conference – IITC.

[73] Nitta S.V., Pisupatti V., Jain A., Wayner P.C., Gill W.N., Plawsky J.L. (1999). Surface modified spin-on xerogel films as interlayer dielectrics. J. Vac. Soc. Technol. B.

[74] Ryan E.T., Ho H.-M., Wu W.-L., Ho P.S., Gidley D.W., Drage J. (1999). Material property characterization and integration issues for mesoporous silica. Proceedings of the IEEE 1999 International Interconnect Technology Conference (Cat. No.99EX247).

[75] Herrmann M., Richter F., Schulz S.E. (2008). Study of nano-mechanical properties for thin porous films through instrumented indentation: SiO_2_ low dielectric constant films as an example. Microelectron. Eng..

[76] Goldblatt R.D., Agarwala B., Anand M.B., Barth E.P., Biery G.A., Chen Z.G., Cohen S., Connolly J.B., Cowley A., Dalton T., Das S.K., Davis C.R., Deutsch A., DeWan C., Edelstein D.C., Emmi P.A., Faltermeier C.G., Fitzsimmons J.A., Hedrick J., Heidenreich J.E., Hu C.K., Hummel J.P., Jones P., Kaltalioglu E., Kastenmeier B.E., Krishnan M., Landers W.F., Liniger E., Liu J., Lustig N.E., Malhotra S., Manger D.K., McGahay V., Mih R., Nye H.A., Purushothaman S., Rathore H.A., Seo S.C., Shaw T.M., Simon A.H., Spooner T.A., Stetter M., Wachnik R.A., Ryan J.G. (2000). A high performance 0.13 μm copper BEOL technology with low-k dielectric. Proceedings of the IEEE 2000 International Interconnect Technology Conference (Cat. No.00EX407).

[77] McGahay V., Adams C., Barth E., Biery G., Chen Z., Chen X., Das S., Davis C., Engel B., Fitzsimmons J., Gill J., Gambino J., Geffken R., Goldblatt R., Hadel L., Kastenmeier B., Klaasen W., Landers W., Luce S., Lustig N., Marino J., Martin A., McDevitt T., McGrath J., Melville I., Nguyen D., Procter R., Rathore H., Seo S.-C., Spooner T., Stamper A., Standaert T., Tian C., Wynne J., McKerrow A.J., Shacham-Diamand Y., Zaima S., Ohba T. (2001). Cu/SiLK integration: influence of process on reliability. Advanced Metallization Conference 2001 (AMC 2001). Proceedings of the Conference.

[78] Strittmatter R.J., Hahnfeld J.L., Silvis H.C., Stokich T.M., Perry J.D., Ouellette K.B., Niu Q.J., Godschalx J.P., Kalantar T.H., Mubarekyan E., Hefner R.E., Lyons J.W., Dominowski J.M., Buske G.R., McKerrow A.J., Leu J., Kraft O., Kikkawa T. (2003). Development of porous SiLK trade semiconductor dielectric resin for the 65 nm and 45 nm nodes. Materials, Technology and Reliability for Advanced Interconnects and Low-k Dielectrics - Symposium 2003.

[79] Baklanov M.R., Mogilnikov K.P. (2002). Non-destructive characterization of low-k dielectric films. Microelectron. Eng..

[80] Lee H.-J., Soles C.L., Liu D.-W., Bauer B.J., Lin E.K., Grill A. (2004). Structural characterization of porous low-k thin films prepared by different techniques using X-ray porosimetry. J. Appl. Phys..

[81] Travaly Y., Sinapi F., Heylen N., Humbert A., Delande M., Caluwaert R., de Mussy J.P., Vereecke G., Baklanov M.R., Iacopi F., Hernandez J.L., Beyer G., Fischer P. (2007). The critical role of the metal/porous low-k interface in post direct CMP defectivity generation and resulting ULK surface and bulk hydrophilisation. 2007 10th International Interconnect Technology Conference.

[82] Bruggeman D.A.G. (1935). Berechnung verschiedener physikalischer Konstanten von heterogenen Substanzen. I. Dielektrizitatskonstanten und Leitfahigkeiten der Mischkorper aus isotropen Substanzen. Annalen der Physik (Leipzig).

[83] Wagh A.S., Poeppel R.B., Singh J.P. (1991). Open pore description of mechanical properties of ceramics. J. Mater. Sci..

[84] Munro R.G. (2004). Analytical representations of elastic moduli data with simultaneous dependence on temperature and porosity. J. Res. NIST.

[85] Liniger E.G., Simonyi E.E. (2004). Moisture-driven crack growth in blanket low dielectric constant and ultralow dielectric constant films. J. Appl. Phys..

[86] Lane M.W., Liu X.H., Shaw T.M. (2004). Environmental effects on cracking and delamination of dielectric films. IEEE Trans. Device Mater. Reliability.

[87] Ryan E.T., Martin H., Junker K., Wetzel J. (2001). Effect of material properties on intgeration damage in organosilicate films. J. Mater. Res..

[88] Worsley M.A., Roberts M., Bent S.F., Gates S.M., Shaw T., Volksen W., Miller R. (2005). Detection of open or closed porosity in low-κ dielectrics by solvent diffusion. Microelectron. Eng..

[89] Kagawa Y., Enomoto Y., Kameshima T., Okomoto M., Kawashima H., Yamada A., Hasegawa T., Akiyama K., Masuda H., Miyajima H., Shibata H., Kadomura S. (2006). Robust 45nm node Cu/ULK interconnects using effective porogen control. Proceedings of the IEEE 2006 International Interconnect Technology Conference (IEEE Cat. No. 06TH8862C).

[90] Caluwaerts R., Van Hove M., Beyer G., Hoofman R.J.O.M., Struyf H., Verheyden G.J.A.M., Waeterloos J., Tokei Zs., Iacopi F., Carbonell L., Le Q.T., Das A., Vos I., Demuynck S., Maex K. (2003). Post patterning meso porosity creation: a potential solution for pore sealing. Proceedings of the IEEE 2003 International Interconnect Technology Conference (Cat. No.03TH8695).

[91] Jousseaume V., Favennec L., Zenasni A., Passemard G. (2006). Plasma-enhanced-chemical-vapor-depositecd ultralow k for a posintegration porogen removal approach. Appl. Phys. Lett..

[92] Ryan E.T., Gates S.M., Grill A., Molis S., Flaitz P., Arnold J., Sankarapandian M., Cohen S.A., Ostrovski Y., Dimitrakopoulos C. (2008). Property modifications of nanoporous pSiCOH dielectrics to enhance resistance to plasma-induced damage. J. Appl. Phys..

[93] Furukawa Y., Patz M., Kokubo T., Snijders J.H.M. (2003). Material modification of the patterned wafer during dry etching and strip determined by XPS. Microelectron. Eng..

[94] Furukawa Y., Wolters R., Roosen H., Snijders J.H.M., Hoofman R. (2004). Etch and strip induced material modofocation of porous low-k (k = 2.2) dielectric. Microelectron. Eng..

[95] Aimadeddine M., Arnal V., Farcy A., Guedj C., Chevolleau T., Possémé N., David T., Assous M., Louveau O., Volpi F., Torres J. (2005). Impact of patterning and ashing on electrical properties and reliability of interconnects in a porous SiOCH ultra low-k dielectric material. Microelectron. Eng..

[96] Dalton T.J., Fuller N., Tweeedie C., Dunn D., Labelle C., Gates S., Colburn M., Chen S.T., Tai L., Dellaguardia R., Petrarca K., Dziobkowski C., Kumar K., Siddiqui S. (2004). Ash-induced modification of porous and dense SiCOH inter-level-dielectric (ILD) materials during damascene plasma processing. Proceedings of the IEEE 2004 International Interconnect Technology Conference (IEEE Cat. No.04TH8729).

[97] Shi H., Bao J., Huang H., Chao B., Smith S., Sun Y., Ho P.S. (2008). Mechanistic study of CO_2_ plasma damage to OSG low k dielectrics. Proceedings of the IEEE 2008 International Interconnect Technology Conference.

[98] Liu H., Widodo J., Liew S.L., Wang Z.H., Wang Y.H., Lin B.F., Wu L.Z., Seet C.S., Low C.H., Liu W.P., Zhou M.S., Hsia L.C. (2009). Challenges of ultra low-k integration in BEOL interconnect for 45nm and beyond. 2009 IEEE International Interconnect Technology Conference – IITC.

[99] Blaschta F., Schulze K., Schulz S.E., Gessner T. (2004). SiO_2_ aerogel ultra low k dielectric patterning using different hard mask concepts and stripping processes. Microelectron. Eng..

[100] White B., Knorr A., Engbrecht W., Kastenmeier B., Das S., McGowan R., Satyanarayana S., Gallagher M. (2005). Dual damascene ash development for a VFTL of target k = 2 integration. Microelectron. Eng..

[101] Grill A., Patel V. (2004). Interaction of hydrogen plasma with extreme low-k SiCOH dielectrics. J. Electrochem. Soc..

[102] Grill A., Sternhagen V., Neumayer D., Patel V. (2005). Hydrogen plasma effects on ultralow-k porous SiCOH dielectrics. J. Appl. Phys..

[103] Grill A., Patel V. (2006). The effect of plasma chemistry on the damage induced to porous SiCOH dielectrics. J. Electrochem. Soc..

[104] Worsley M.A., Bent S.F., Gates S.M., Fuller N.C.M., Volksen W., Steen M., Dalton T. (2005). Effect of plasma interactions with low-κ films as a function of poroity, plasma chemistry, and temperature. J. Vac. Sci. Technol. B..

[105] Blaschta F., Schulz S.E., Gessner T. (2005). Impact of resist stripping processes at elevated temperature on ULK and HM materials. Microelectron. Eng..

[106] Broussou L., Puyrenier W., Rebiscoul D., Rouessac V., Ayral A. (2008). Post-etch cleaning for porous low-k integration: impact of HF wet etch on “pore-sealing” and “k recovery”. Proceedings of the IEEE 2008 International Interconnect Technology Conference.

[107] Abell T., Maex K. (2004). Damage minimized plasma pore sealing of microporous low k dielectrics. Microelectron. Eng..

[108] Hoyas A.M., Schumacher J., Whelan C.M., Celis J.P., Maex K. (2004). Plasma sealing of a low-K dielectric polymer. Microelectron. Eng..

[109] Puyrenier W., Rouessac V., Broussou L., Rébiscoul D., Ayral A. (2006). Characterization of the impact of plasma treatments and wet cleaning on a porous low k material. Microelectron. Eng..

[110] Guo H.-W., Zhu L., Zhang L., Ding S.-J., Zhang D.W., Liu R. (2008). Influence of NH3 plasma treatment on chemical bonding and water adsorption of low-k SiCOH film. Microelectron. Eng..

[111] Broussou L., Puyrenier W., Rebiscoul D., Rouessac V., Ayral A. (2007). Porosity and structure evolution of a SiOCH low k material during post-etch cleaning process. Microelectron. Eng..

[112] Arakawa S., Mizuno I., Ohoka Y., Nagahata K., Tabuchi K., Kanamura R., Kadomura S. (2006). Breakthrough integration of 32nm node Cu/ultra low-k SiOC (k-2.0) interconnects by using advanced pore-sealing and low-k hardmask technologies. Proceedings of the IEEE 2006 International Interconnect Technology Conference (IEEE Cat. No. 06TH8862C).

[113] Sun J.-N., Gidley D.W., Dull T.L., Frieze W.E., Yee A.F., Ryan E.T., Lin S., Wetzel J. (2001). Probing diffusion barrier integrity on porous silica low-k films using positron annihilation lifetime spectroscopy. J. Appl. Phys..

[114] Jezewski C., Wiegand C.J., Ye D., Mallikarjunan A., Liu D., Jin C., Lanford W.A., Wang G.-C., Senkevich J.J., Lu T.-M. (2004). Molecular caulking: a pore sealing CVD polymer for ultralow k dielectrics. J. Electrochem. Soc..

[115] Ou Y., Wang P.-I., Vanamurthy L.H., Bakhru H., Lu T.-M., Spencer G. (2008). Thermal stability study of pore sealing using Parylene N. J. Electrochem. Soc..

[116] Xie B., Muscat A.J. (2004). Silylation of porous methylsilsesquioxane films in supercritical carbon dioxide. Microelectron. Eng..

[117] Xie B., Muscat A.J. (2005). The restoration of porous methylsilsesquioxane (p-MSQ) films using trimethylhalosilanes dissolved in supercritical carbon dioxide. Microelectron. Eng..

[118] Chaabouni H., Chapelon L.L., Aimadeddine M., Vitiello J., Farcy A., Delsol R., Brun P., Fossati D., Arnal V., Chevolleau T., Joubert O., Torres J. (2007). Sidewall restoration of porous ultr low-k dielectrics for sub-45nm technology nodes. Microelectron. Eng..

[119] Yamanishi T., Ono T., Kohmura K., Fujii N., Nakatama T., Tanaka H., Chikaki S., Kikkawa T. (2007). Removal of etching/ashing residues and ashing/wet-clean damage in porous silica low-k films. Microelectron. Eng..

[120] Oszinda T., Schaller M., Fischer D., Schulz S.E. (2009). Characterization of plasma damaged porous ULK SiCOH layers in aspect of changes in the diffusion behavior of solvents and repair chemicals. 2009 IEEE International Interconnect Technology Conference – IITC.

[121] Bao J.J., Shi H.L., Liu J.J., Huang H., Ho P.S., Goodner M.D., Moinpour M., Kloster G.M. (2007). Mechanistic study of plasma damage and CH4 recovery of low k dielectric surface. 2007 10th International Interconnect Technology Conference.

[122] Shaw T.M., Liniger E., Bonilla G., Doyle J.P., Herbst B., Liu X.H., Lane M.W. (2007). Experimental determination of the toughness of crack stop structures. 2007 10th International Interconnect Technology Conference.

[123] Kearney A.V., Vairagar A.V., Geisler H., Zschech E., Dauskardt R.H. (2007). Assesing the effect of die sealing in Cu/low-k structures. 2007 10th International Interconnect Technology Conference.

[124] Tsuda H., Kageyama S., Katayama S., Ohashi N., Matsubara Y., Kobayashi N. (2004). Suppression of Cu Extrusion into porous-MSQ film during chip-reliability test. Proceedings of the IEEE 2004 International Interconnect Technology Conference (IEEE Cat. No.04TH8729).

[125] Ong J., Zhang X., Kripesh V., Lim Y.K., Yeo D., Chan K.C., Tan J.B., Hsia L.C., Sohn D.K., Tay A. (2007). Structural design and optimization of 65nm Cu/low-k flipchip package. 2007 9^th^ Electronics Packaging Technology Conference.

[126] Gates S.M., Dubois G., Ryan E.T., Grill A., Liu M., Gidley D. (2009). Adjusting the skeleton and pore structure of porous SiCOH dielectrics. J. Electrochem. Soc..

[127] Dubois G., Volksen W., Magbitang T., Sherwood M.H., Miller R.D., Gage D.M., Dauskardt R.H. (2008). Superior mechanical properties of dense and porous organic/inorganic hybrid thin films. J. Sol-Gel Sci. Technol..

[128] Torquato S., Donev A., Evans A.G., Brinker C.J. (2005). Manufacturable extremal low-dielectric, high-stiffness porous materials. J. Appl. Phys..

[129] Hata N., Negoro C., Yamada K., Kikkawa T. (2004). Control of pore structures in periodic porous silica low-k films. Jpn. J. Appl. Phys..

[130] Nitta S., Edelstein D., Ponoth S., Clevenger L., Liu X., Standaert T. (2008). Performance and reliability of airgaps for advanced BEOL interconnects. Proceedings of the IEEE 2008 International Interconnect Technology Conference.

